# P-1330. Knowledge of Lyme Disease and Prevention Practices Among Parents of Pediatric Patients in a Lyme-Endemic Area

**DOI:** 10.1093/ofid/ofae631.1508

**Published:** 2025-01-29

**Authors:** Sheila Krishnan, Andrew S Handel

**Affiliations:** Stony Brook Children's Hospital, Stony Brook, New York; Stony Brook Children's Hospital, Stony Brook, New York

## Abstract

**Background:**

Lyme disease (LD) is a growing public health concern in the US that disproportionately affects children and the elderly. Studies have also found that minority groups are more likely to present with later, severe manifestations of LD, suggesting missed opportunities for early diagnosis and treatment. Despite the prevalence, few assessments have been performed to measure tickborne disease knowledge among parents in endemic areas, including social determinants of health.

**Table 1.**
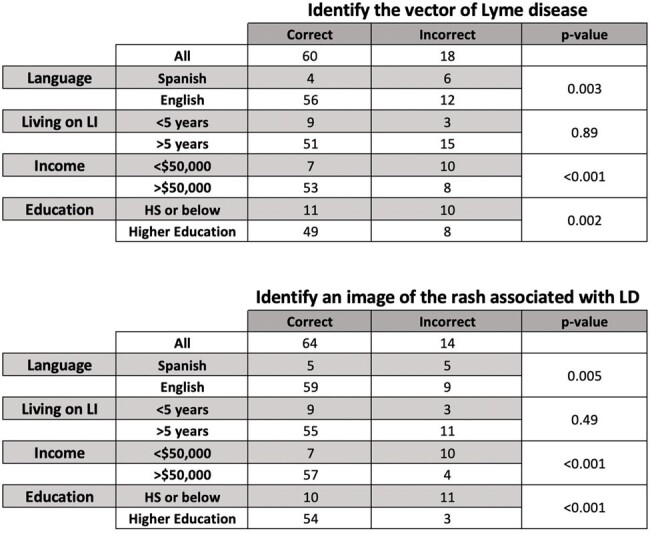
Respondents with Spanish language preference, household income <$50,000, and highest education level of high school or below were more likely to misidentify the vector of Lyme disease or an image of the EM rash

**Methods:**

An anonymous survey was conducted among parents/caregivers of patients at Stony Brook Children’s Hospital and its six ambulatory clinics in Long Island, NY (LI). The study was approved by the Stony Brook University Hospital IRB. Flyers were posted in patient rooms with QR codes linking to a Qualtrics™-based study, which displayed the informed consent statement and survey instrument. The flyers and survey were available in English and Spanish. Survey questions included demographics (income, level of education, etc), knowledge assessment (including identification of ticks as a vector and the erythema migrans (EM) rash), and tick bite prevention practices. Proportions were compared using χ^2^ tests.

**Results:**

78 respondents completed the survey, including 68 (87%) in English and 10 (13%) in Spanish. 12 (15%) reported living on LI for < 5 years, and 17 (22%) reported household income < $50,000, while 7 (9%) reported household income below $20,000. 21 (27%) reported highest level of education as high school. 18 (26%) did not correctly identify ticks as the vector for LD, while 14 (21%) did not correctly identify an EM. Respondents with Spanish preference, household income < $50,000, and highest education level of high school or below were more likely to misidentify the vector of Lyme disease or an EM image (Table 1).

**Conclusion:**

Knowledge of LD remains limited, even in endemic areas. This is more pronounced in those with lower income, education, and Spanish preference. Culturally and linguistically appropriate public health outreach should focus on education about disease transmission and early recognition.

**Disclosures:**

**All Authors**: No reported disclosures

